# Long-term effects of peginterferon alfa-2a therapy in Japanese patients with chronic hepatitis B virus infection

**DOI:** 10.1186/s12985-015-0453-7

**Published:** 2015-12-23

**Authors:** Keiichi Masaki, Fumitaka Suzuki, Tasuku Hara, Yusuke Kawamura, Hitomi Sezaki, Tetsuya Hosaka, Norio Akuta, Masahiro Kobayashi, Satoshi Saitoh, Yoshiyuki Suzuki, Yasuji Arase, Kenji Ikeda, Mariko Kobayashi, Hiromitsu Kumada

**Affiliations:** Department of Hepatology, Toranomon Hospital, Tokyo, Japan; Research Institute for Hepatology, Toranomon Hospital, Tokyo, Japan

**Keywords:** Peginterferon alfa-2a, Hepatitis B virus, Chronic hepatitis B, Genotype, Hepatitis B surface antigen

## Abstract

**Background:**

There is no information on the long-term effects of peginterferon (PEG-IFN) alfa-2a therapy for chronic hepatitis B (CHB) in Japan. This double-blind, randomized trial investigated the efficacy of PEG-IFN therapy.

**Methods:**

We analyzed 22 Japanese patients with CHB (hepatitis B e antigen [HBeAg]-positive: 17, HBeAg-negative: 5) treated with PEG-IFN alfa-2a and followed-up posttreatment for 5 years. Responders represented patients who showed persistent normalization of alanine transferase (ALT) levels, HBeAg clearance, and low hepatitis B virus (HBV) DNA levels (HBeAg-positive patient; <5 log copies/mL, HBeAg-negative patient; <4.3 log copies/mL) at end of treatment, and at 1, 2, 3, 4 and 5 years posttreatment. In addition, baseline HBeAg-positive patients who showed sustained normalization of ALT level, HBeAg clearance, and low HBV DNA level for more than 6 months until at 1, 2, 3, 4, and 5 years after completion of PEG-IFN were also classified as “triple responders” and the proportion of triple responders relative to all patients was termed the “triple response rate”.

**Results:**

The response rates among HBeAg-positive patients were 13 %, 25 %, 14 %, 21 % and 21 % at end of treatment, and at 1, 2, 3, 4, and 5 years, respectively. The response rate tended to be higher in patients treated for 48 than 24 weeks. The respective response rates among HBeAg-negative patients were 0 %, 20 %, 20 %, 20 % and 25 %. During the treatment period, hepatitis B surface antigen (HBsAg) clearance at 3.5 years was noted in one patient, who was 37-year-old, male, had genotype C and received PEG-IFN alfa-2a at 90 μg for 48 weeks.

**Conclusion:**

At 5 years after completion of PEG-IFN, the triple response rate in HBeAg-positive patients and combined response rate in HBeAg-negative patients were 21 % (3/14) and 25 % (1/4), respectively. The triple response was seen in three patients who had all been treated with PEG-IFN for 48 weeks.

## Background

Hepatitis B virus (HBV) infection is a common disease that can induce chronic carrier state and is associated with risk of development of progressive disease and hepatocellular carcinoma [1]. Interferon (IFN) and several nucleoside/nucleotide analogues, such as lamivudine, adefovir dipivoxil, entecavir, and tenofovir disoproxil fumarate, are currently approved for the treatment of chronic hepatitis B (CHB) in several countries [2–8]. These drugs can suppress the activity of hepatitis and progression of liver fibrosis, thus preventing hepatic failure and hepatocarcinogenesis and improving quality of life and survival [9–11]. Successful treatment of CHB with clearance of hepatitis B e antigen (HBeAg), reduction in serum HBV DNA levels, and normalization of alanine transferase (ALT) levels is associated with favorable long-term outcome, independent of the antiviral drug used [12].

However, conventional IFN alfa has suboptimal pharmacokinetics, and it requires administration three times a week, resulting in fluctuating drug exposure and burden on patients [3, 4]. On the other hand, one of the problems associated with the use of nucleoside/nucleotide analogues is the low HBeAg seroconversion rate [6, 13]. Furthermore, protracted use of nucleoside/nucleotide analogues, such as lamivudine or adefovir dipivoxil, can increase the likelihood of drug resistance [4, 5, 14] and late adverse effects [15].

Peginterferon (PEG-IFN) alfa-2a, prepared by attaching a large, branched, 40-kD polyethylene glycol molecule to IFN alfa-2a [16], has better pharmacokinetics than the conventional IFN alfa, allowing once-weekly dosing and maintenance of effective serum concentrations throughout the dosage interval [17]. A randomized study of PEG-IFN alfa-2a therapy published in 2004 involving 537 HBeAg-negative adult patients treated for 48 weeks with either PEG-IFN alfa-2a or lamivudine, or a combination of the two, reported significantly higher posttreatment response rates with PEG-IFN alfa-2a compared with lamivudine alone. Furthermore, ALT normalization at 6 months posttreatment was noted in 59 % of patients treated with PEG-IFN alfa-2a, 60 % of patients treated with the combination of PEG-IFN alfa-2a and lamivudine, and 44 % of patients treated with lamivudine alone (*P* = 0.004, *P* = 0.003), with 43 %, 44 %, 29 % rates of HBV DNA levels of ≤20,000 copies/ml at 6 months posttreatment, respectively (*P* = 0.007, *P* = 0.003) [18]. Another randomized study of 814 HBeAg-positive adult patients treated for 48 weeks with either PEG-IFN alfa-2a or lamivudine, or their combination published in 2004 showed significantly higher posttreatment response rate for PEG-IFN alfa-2a compared with lamivudine. HBV DNA level was ≤100,000 copies/ml at 6 months posttreatment in 32 % of patients treated with PEG-IFN alfa-2a, 34 % of those treated with the combination of PEG-IFN alfa-2a and lamivudine, and 22 % of those treated with lamivudine alone (*P* = 0.012, *P* = 0.003), with HBeAg seroconversion rates at 6 months posttreatment of 32 %, 27 %, 19 %, respectively (*P* < 0.001, *P* = 0.002) [19].

Another long-term follow-up study of 315 of patients treated with either PEG-IFN alfa-2a or lamivudine, or their combination showed a higher rate of ALT normalization at 3 years posttreatment in patients treated with PEG-IFN alfa-2a (31 %) than with lamivudine alone (18 %, *P* = 0.032), and HBV DNA levels of ≤10,000 copies/ml were noted in 28 % and 15 % of the patients, respectively (*P* = 0.039). In addition, 8.7 % of those treated with PEG-IFN alfa-2a-containing regimen showed HBsAg clearance [20].

However, specific data on the long-term effects of PEG-IFN therapy particularly for Japanese patients are not available at present. The aim of the present study was to determine the long-term effects of PEG-IFN alfa-2a administered for 24 or 48 weeks, at dose of 90 and 180 μg in Japanese patients with CHB.

## Methods

### Patients

This double-blind, randomized trial included 22 Japanese patients (6 females and 16 males) who were chronically infected with HBV alone (confirmed HBsAg positivity for at least six months and negativity for other hepatitis viruses) and commenced PEG-IFN treatment between September 2007 and December 2008 at the Department of Hepatology, Toranomon Hospital (Table 1). Patients were included in this study if they aged more than 20 years, serum HBsAg-positive, HBsAb-negative by screening, high ALT twice within 180 days of enrolment, and confirmed CHB. In HBeAg-positive patients, the following criteria were also used for inclusion; positivity for DNA polymerase, with HBV DNA ≥5.7 log copies/ml. The following criteria were also used for inclusion of HBeAg-negative patients; negativity for HBeAg, with HBV DNA ≥5.0 log copies/ml. All patients were confirmed to have HBV hepatitis and not other vectors, such as infection with hepatitis C virus or autoimmune hepatitis. None had history of drug abuse or alcoholic hepatitis. The exclusion criteria were neutrophil count <1,500/mm^3^, platelet count < 90,000/mm^3^, hemoglobin concentration <10 g/dL, decompensated liver disease, history of organ transplantation, creatinine clearance <50 ml/min, severe psychiatric disease, diabetes requiring pharmacotherapy, poorly controlled hypertension, malignancy, severe or chronic cardiac or pulmonary disease, immunologically-mediated disease, stroke or retinopathy and previous treatment with pegylated INF.

**Table 1:** Characteristics of patients at commencement of interferon therapy

Demographic data	
n	22
Sex, (female/male)	6/16
Age, years (range)	33 (22–50)
Previous treatment with interferon	6 (27.2 %)
Previous treatment with nucleoside analog	1 (1.7 %)
Laboratory Data	
Aspartate aminotransferase, IU/L (range)	58 (28–237)
Alanine aminotransferase, IU/L (range)	111 (41–397)
Bilirubin, mg/dL (range)	0.7 (0.3-1.6)
Albumin, g/dL (range)	3.9 (3.5-4.4)
Platelets, ×10^3^/μL (range)	196 (106–306)
HBsAg, (IU/ml)	2370 (22.1-75500)
Serum HBV DNA, log copies/mL (range)	7.6 (5.3 to >7.6)
HBeAg, positive/negative	17/5
HBV genotype (A/B/C)	2/2/18

Data are median and (range) or number and (percentage)

*HBV* hepatitis B virus, *HBeAg* Hepatitis B e antigen, *ND* not done

The study was conducted in accordance with the ethical principles of the Declaration of Helsinki and was approved by the Toranomon Hospital Ethical Committee. Informed consent was obtained from each patient.

### Interferon therapy and assessment of response to therapy

Of the 22 patients included in this study, 17 were HBeAg-positive and 5 were HBeAg-negative. The 17 HBeAg-positive patients were randomly assigned to receive PEG-IFN alfa-2a (PEGASYS: Hoffmann-La Roche Inc., Basel, Switzerland) at either 90 μg for 24 weeks (*n* = 4) or 48 weeks (*n* = 4), 180 μg for 24 weeks (*n* = 5), or 48 weeks (*n* = 4). The 5 HBeAg-negative patients were randomly assigned to receive PEG-IFN alfa-2a at either 90 μg (*n* = 3) or 180 μg (*n* = 2) for 48 weeks.

The number of responders was evaluated at the end of each treatment, and at 0.5, 1, 2, 3, 4, and 5 years after completion of PEG-IFN alfa-2a. Among the HBeAg-positive patients at baseline, responders were defined as patients who showed normalization of serum ALT level (normal level: 6–30 IU/L), HBeAg clearance, and low HBV DNA level (<5 log copies/mL) at the end of completion of PEG-IFN alfa-2a treatment. In addition, baseline HBeAg-positive patients who showed sustained normalization of ALT level, HBeAg clearance, and low HBV DNA level for more than 6 months until each point at 0.5, 1, 2, 3, 4, and 5 years after completion of PEG-IFN alfa-2a were also classified as “triple responders” and the proportion of triple responders relative to all patients was termed the “triple response rate”. Among baseline HBeAg-negative patients, responders were defined as those who showed sustained normalization of ALT level and low HBV DNA level (<4.3 log copies/mL) for more than 6 months until each point after completion of PEG-IFN alfa-2a therapy. In addition, baseline HBeAg-negative patients who showed sustained normalization of ALT level and low HBV DNA level for more than 6 months until each point at 0.5, 1, 2, 3, 4, and 5 years after completion of PEG-IFN alfa-2a were also classified as “Combined responders”.

All patients not considered responders were labeled as “non-responders.” Patients who were switched to other therapies (IFN or nucleoside/nucleotide analogues) after completion of the study were also considered non-responders.

### Blood tests and serum viral markers

Routine biochemical tests were performed monthly using standard procedures during treatment and the first 12 months following IFN therapy and at least every 2 months thereafter. Levels of HBsAg, HBeAg, and anti-HBe were determined using enzyme-linked immunosorbent assay (ELISA) using a commercial kit (HBeAg EIA; Institute of Immunology, Tokyo) or Chemiluminescent Enzyme Immunoassay (CLEIA; Lumipulse System, Fujirebio, Inc. Tokyo). HBV DNA levels were measured using COBAS TaqMan HBV Test; v1.0 or v2.0, (Roche Diagnostics K.K., Tokyo).

### HBV genotype

The major genotypes of HBV were determined using ELISA (Institute of Immunology, Tokyo) or PCR-invader assay (BML, Inc) according to the method described by Usuda et al. [21] and Tadokoro et al. [22].

## Results

### Study population

The majority of patients were male (*n* = 16, 73 %), and infected with HBV genotype C (*n* = 18, 82 %). The baseline characteristics of the patients are summarized in Table 1. Although few patients had genotypes A and B, the distribution of HBV genotype was similar in patients with CHB who received care in our hospital, with a follow-up period of more than 2 years [23]. Two of 2 patients with genotype A, 1 of 2 with genotype B, 14 of 18 with genotype C were HBeAg-positive at the commencement of treatment. At 5 years after completion of PEG-IFN, 11 patients in HBeAg-positive case, and 4 patients in HBeAg-negative case were switched to other treatments. Three HBeAg-positive patients did not complete the follow-up. Eleven patients of the HBeAg-positive group were switched to another treatment (5 patients: entecavir, 6 patients: conventional IFN alfa therapy) from one to four years after PEG-IFN alfa-2a. Four patients of the HBeAg-negative group were switched to another treatment (2 patients: entecavir, 2 patients: conventional IFN alfa therapy) from one to two years after PEG-IFN alfa-2a. Adverse events, such as diarrhea and neutropenia, were noted in some of the patients, but there were no serious adverse events that required cessation of treatment and all patients completed the study without discontinuation of the allocated therapy.

### Response of HBeAg-positive patients to PEG-IFN alfa-2a therapy

ALT normalization among HBeAg-positive patients was noted in 12 % (2/17), 29 % (5/17), 19 % (3/16), 31 % (5/16), 21 % (3/14), 21 %(3/14) and 21 % (3/14) at the end of treatment, and at 0.5, 1, 2, 3, 4, and 5 years after completion of PEG-IFN alfa-2a, respectively. The rate of HBeAg seroconversion was 24 % (4/17), 24 % (4/17), 25 % (4/16), 25 % (4/16), 21 % (3/14), 21 % (3/14) and 21 % (3/14), respectively. The rate of low HBV level (<5 log copies/mL) was 41 % (7/17), 18 % (3/17), 19 % (3/16), 31 % (5/16), 21 % (3/14), 21 % (3/14) and 21 % (3/14), respectively (Table 2). The triple response rate was 6 % (1/17), 6 % (1/17), 13 % (2/16), 25 % (4/16), 14 % (2/14), 21 % (3/14) and 21 % (3/14), respectively. Among the patients who achieved the triple response at 2 years, one patient did not complete the follow-up and another patient had ALT rebound (up to 32 IU/L). Two of 3 patients who achieved triple response at 5 years after completion of PEG-IFN were genotype C and the other was genotype A. At more than 2 years after completion of PEG-IFN alfa-2a, the rates of ALT normalization, HBeAg seroconversion, low HBV level (<5 log copies/mL), and triple response rate tended to be higher in patients treated for 48 weeks with PEG-IFN alfa-2a than 24 weeks. The three patients who achieved triple response were young (range, 26–34 years), had high titer of ALT, low titer of HBeAg, and received PEG-IFN alfa-2a treatment for 48 weeks (Table 3).

**Table 2:** Response of HBeAg-positive patients to various doses and duration of PEG-IFN alfa-2a therapy analyzed according to endpoint

Dose/Duration and endpoint	EOT	Time after end of treatment (years)					
		0.5	1	2	3	4	5
90 μg / 24 week							
ALT normalization	1/4	0/4	0/4	0/4	0/4	0/4	0/4
HBeAg seroconversion	0/4	0/4	0/4	0/4	0/4	0/4	0/4
Low HBV (<5 log copies/mL)	0/4	0/4	0/4	0/4	0/4	0/4	0/4
180 μg / 24 week							
ALT normalization	0/4	0/4	0/4	1/4	0/3	0/3	0/3
HBeAg seroconversion	0/4	0/4	1/4	1/4	0/3	0/3	0/3
Low HBV (<5 log copies/mL)	2/4	0/4	1/4	2/4	0/3	0/3	0/3
90 μg/48 week							
ALT normalization	0/4	2/4	1/3	2/3	1/3	2/3	2/3
HBeAg seroconversion	1/4	1/4	1/3	2/3	2/3	2/3	2/3
Low HBV (<5 log copies/mL)	1/4	2/4	1/3	2/3	2/3	2/3	2/3
180 μg/48 week							
ALT normalization	1/5	3/5	2/5	2/5	2/4	1/4	1/4
HBeAg seroconversion	3/5	3/5	2/5	1/5	1/4	1/4	1/4
Low HBV (<5 log copies/mL)	4/5	1/5	1/5	1/5	1/4	1/4	1/4

*EOT* end of treatment

**Table 3:** Clinical features of three patients who achieved triple response

Number	age (years)/sex	genotype	Pretreatment HBV titer (log copies/mL)	Pretreatment HBsAg titer (IU/ml)	HBeAg	Dose of PEG-IFN (μg)	Duration of PEG-IFN treatment (weeks)	Pretreatment AST/ALT (IU/ml)
1	26/male	C	7.6	4470	positive	180	48	47/123
2	30/female	C	5.3	1000	positive	90	48	45/62
3	34/male	A	7.6	881	positive	90	48	37/397

### Response of HBeAg-negative patients to PEG-IFN alfa-2a therapy

ALT normalization among HBeAg-negative patients was noted in 40 % (2/5), 60 % (3/5), 60 % (3/6), 20 % (1/5), 20 % (1/5), 20 % (1/5) and 25 % (1/4) at the end of treatment, and at 0.5, 1, 2, 3, 4, and 5 years after completion of PEG-IFN alfa-2a, respectively. Furthermore, the respective rates of low HBV level (<4.3 log copies/mL) were 80 % (4/5), 20 % (1/5), 0 % (0/5), 20 % (1/5), 20 % (1/5), 20 % (1/5) and 25 % (1/4) (Table 4). The combined response rate was 40 % (2/5), 20 % (1/5), 0 % (0/5), 20 % (1/5), 20 % (1/5), 20 % (1/5), and 25 % (1/4), respectively. The single patient who achieved combined response at 3 years after completion of PEG-IFN was genotype C. Only one patient who received PEG-IFN alfa-2a at 90 μg for 48 weeks achieved ALT normalization and low HBV level (<4.3 log copies/mL) at more than 2 years after completion of PEG-IFN.

**Table 4:** Response of HBeAg-negative patients to various doses and duration of PEG-IFN alfa-2a therapy analyzed according to endpoint

Dose/duration and endpoint	EOT	Time after end of treatment (years)					
		0.5	1	2	3	4	5
90 μg/48 week							
ALT normalization	1/3	1/3	1/3	1/3	1/3	1/3	1/2
Low HBV (<4.3 log copies/mL)	2/3	1/3	0/3	1/3	1/3	1/3	1/2
180 μg/48 week							
ALT normalization	1/2	2/2	2/2	0/2	0/2	0/2	0/2
Low HBV (<4.3 log copies/mL)	2/2	0/2	0/2	0/2	0/2	0/2	0/2

*EOT* end of treatment

### HBsAg seroclearance

**Fig. 1:**
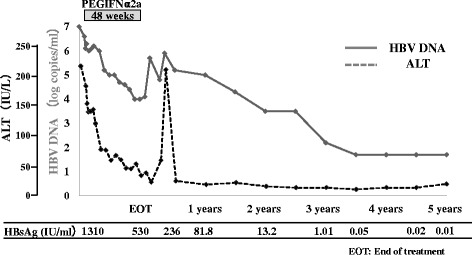
Clinical course of the patient who achieved HBsAg loss. EOT: End of treatment

At 3.5 years, HBsAg clearance occurred in one male patient who was 37 years old, had genotype C and was treated with PEG-IFN alfa-2a at 90 μg for 48 weeks. In this patient, ALT and HBV-DNA levels decreased during the treatment period (ALT: from 182 at baseline to 38 IU/ml, HBV-DNA: from 6.6 to 4.1 log copies/ml, respectively). However, their levels gradually increased from the end of treatment to 8 weeks posttreatment (ALT: from 38 to 209 IU/ml, HBV-DNA: from 4.1 to 5.9 log copies/ml), but then decreased again up to 5 years (ALT: 20 IU/ml, HBV-DNA: <1.5 log copies/ml) [Fig. 1]. None of the other patients showed HBsAg seroclearance.

### ALT levels during follow-up

**Fig. 2:**
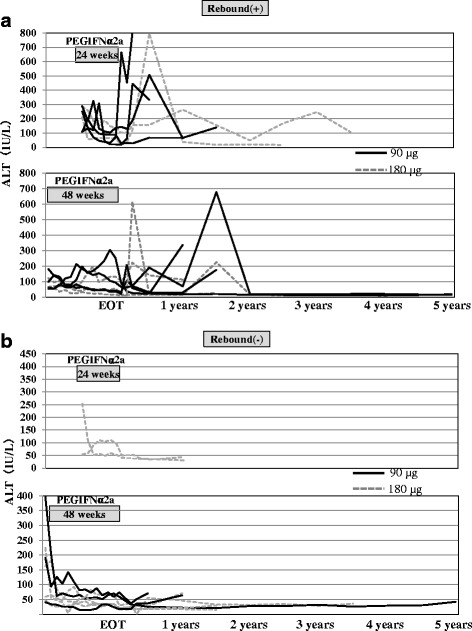
ALT levels of individual patients from pretreatment to end of treatment, and at 0.5, 1, 2, 3, 4 and 5 years after completion of treatment according to the duration of PEG-IFN treatment **a** with ALT levels rebound, **b** without ALT levels rebound. EOT: End of treatment

We defined ALT rebound as elevation of ALT level at posttreatment to ≥100 IU/ml than the nadir. Figure 2a and b show ALT levels during and after PEG-IFN alfa-2a treatment with and without ALT rebound. Among the 17 HBeAg-positive patients, 10 showed ALT rebound, while 3 of the 5 (60 %) HBeAg-negative patients showed ALT rebound. Among these 10 patients, one of six (17 %) patients who received PEG-IFN alfa-2a for 24 weeks, achieved HBeAg seroconversion and had persistently low HBV DNA level (<5 log copies/mL). Two of 4 (50 %) patients who received PEG-IFN alfa-2a for 48 weeks achieved HBeAg seroconversion and had persistently low HBV levels (<5 log copies/mL). On the other hand, among HBeAg-positive patients who did not show ALT rebound, one of 2 patients who received PEG-IFN alfa-2a for 24 weeks had persistently low HBV level (<5 log copies/mL), and two of the 5 (40 %) patients who received PEG-IFN alfa-2a for 48 weeks achieved HBeAg seroconversion and had persistently low HBV levels (<5 log copies/mL). The baseline ALT levels of the patients who did not show ALT rebound and achieved HBeAg seroconversion were higher than 200 IU/L. Among the HBeAg-negative patients, 3 of the 5 patients showed ALT rebound and had low HBV DNA levels.

### Clinical course

**Fig. 3:**
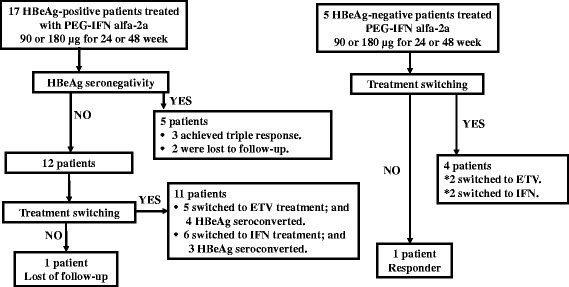
Schematic diagram of the clinical course of study participants including HBeAg-positive and HBe-negative patients

The clinical course is illustrated in Fig. 3. Among the 17 HBeAg-positive patients, 5 achieved HBeAg seroconversion (3 patients during treatment and 2 posttreatment; 1 at 1 year posttreatment and 1 at 2 year posttreatment). Three of the 5 HBeAg seroconverted patients achieved triple response. Among the 12 patients who did not achieve HBeAg seroconversion, 11 were switched to another treatment (5 patients: entecavir, 6 patients: conventional IFN alfa therapy). Specifically, of the 4 patients who were treated with PEG-IFN alfa-2a at 90 μg for 24 weeks, 1 and 3 were switched to entecavir and conventional IFN, respectively. Furthermore, among the 3 patients who received PEG-IFN alfa-2a at 180 μg for 24 weeks, 1 and 2 were switched to entecavir and conventional IFN, respectively. The single patient who received PEG-IFN alfa-2a at 90 μg for 48 weeks was switched to conventional IFN and the 3 patients who received PEG-IFN alfa-2a at 180 μg for 48 weeks were switched to entecavir. Among them, HBeAg seroconversion was noted in 4 of the 5 patients who were treated with entecavir and 3 of 6 patients who received IFN therapy. Among HBeAg-negative patients, 4 of 5 patients were switched to another treatment; entecavir (*n* = 2), conventional IFN alfa therapy (*n* = 2). Furthermore, those who received PEG-IFN alfa-2a at 90 μg for 48 weeks, 2 were switched to entecavir and 2 to conventional IFN. The two patients who received PEG-IFN alfa-2a at 180 μg for 48 weeks were switched to entecavir (*n* = 1) and conventional IFN (*n* = 1). All patients who were switched to NAs treatment had low HBV levels (under the measurement range) during treatment with nucleoside analogs (NA).

## Discussion

The goal of antiviral therapy in CHB is to prevent liver failure and hepatocarcinogenesis by suppressing the activity of hepatitis, progression of liver fibrosis, and improvement of life expectancy and quality of life. The long-term goal of this therapy is elimination of HBsAg. On the other hand, the short-term goals of antiviral treatment are persistent normalization of ALT, HBeAg negativity, and suppression of HBV-DNA replication.

PEG-IFN alfa-2a, like conventional IFN alfa, has a dual immunomodulatory and antiviral modes of action. In Japan, since 1988, 4-week treatment with IFN is fully covered by the Government-based universal healthcare system. Furthermore, since 2002, 24-week treatment, and since 2011, 48-week PEG-IFN treatment were introduced. One randomized study [24] reported an increase in triple response (HBeAg seroconversion, HBV DNA <5 log copies/ml and ALT normalization) rate at 24 weeks post-treatment in HBeAg-positive patients, and that such response correlated with the dose and duration of treatment (PEG-IFN alfa-2a at 90 μg for 24 weeks: 4.9 %, PEG-IFN alfa-2a at 180 μg for 24 weeks: 9.8 %, PEG-IFN alfa-2a at 90 μg for 48 weeks: 17.1 %, PEG-IFN alfa-2a at 180 μg for 48 weeks:19.5 %). Among HBeAg-negative patients, treatment with PEG-IFN alfa-2a at 90 μg and 180 μg for 48 weeks resulted in 37.5 % and 37.9 % HBV DNA suppression (<4.3 log copies/ml at 24 weeks posttreatment), respectively. However, there are no data on the long-term response to PEG-IFN alfa-2a treatment in Japanese patients with CHB, and thus the present study is the first that documents the long-term outcome of treatment with PEG-IFN alfa-2a in Japanese patients with CHB.

We reported in a previous study that HBeAg, HBV-DNA level, age, sex, IFN pretreatment, duration of treatment, and levels of albumin and aspartate transaminase (AST) were important determinants of the long-term response to IFN, and that non-C genotype was an important factor in HBeAg-positive patients [2]. In the NEPTUNE study, Liaw et al. [25] reported that treatment with PEG-IFN alfa-2a at 180 μg for 48 weeks was the most efficacious and beneficial treatment regimen for HBeAg-positive patients predominantly infected with genotypes B or C, compared with lower doses and shorter durations. Following that study, PEG-IFN alfa-2a at 180 μg for 48 weeks became the standard method of treatment for CHB in Europe and the US [9, 26]. Previous studies also reported that older age [27, 28], female sex [27], high titer of ALT [24], low HBV-DNA levels [19, 25, 27, 28], HBsAg [25], genotype A (compared to genotype D) [27, 28], and IL28B (major) [28] also influenced the response to PEG-IFN alfa-2a therapy in HBeAg-positive patients. Furthermore, younger age [29], female sex [29], high titer of ALT [20, 29], low HBV-DNA levels [29], genotypes B or C (compared to genotype D) [29] influenced the response to PEG-IFN therapy in HBeAg-negative patients. In the present study, triple response was noted in three HBeAg-positive patients treated for 48 weeks. These patients were young (range 26–34 years), had high titers of ALT, low titers of HBeAg, and received PEG-IFN alfa-2a treatment for 48 weeks. These findings are similar to those reported in the above previous studies, although there were no dose-related differences. Further, in the present study, two of 3 patients were males, which was different from the above previous studies. The reason for the different finding was considered the small sample size, especially only one female received PEG-IFN alfa-2a treatment for 48 weeks.

Marcellin et al. [17] reported that persistently low level of HBV-DNA is associated with HBsAg clearance during the posttreatment period. In the present study, 3 of 14 (21 %) HBeAg-positive patients achieved low HBV levels (<5 log copies/mL) at 5 years after completion of PEG-IFN, and could potentially show HBsAg clearance. HBsAg clearance was observed in one HBeAg-negative patient treated with PEG-IFN alfa-2a at 90 μg for 48 weeks. HBV-DNA levels in this patient decreased during the treatment period, and gradually decreased after the rebound up to 5 years. In this patient, the low HBV-DNA level and HBsAg clearance during the posttreatment period were considered to be due to the sustained long-term effects of PEG-IFN alfa-2a. We defined ALT rebound at posttreatment as elevated ALT to ≥100 IU/ml than the nadir. We examined ALT levels in patients with [Fig. 2a] and without [Fig. 2b] rebound for each dose and treatment duration. ALT flares during IFN therapy were reported to be associated with high virological response [30]. In the present study, HBeAg-positive patients with ALT rebound achieved higher rate of HBeAg seroconversion and low HBV levels (<5 log copies/mL) among those treated with PEG-IFN alfa-2a for 48 weeks compared to those who were treated for 24 weeks. This finding was considered to be due to changes in host immune response to HBV resulting from the immunomodulating effects of PEG-IFN.

PEG-IFN therapy for a finite duration may provide drug-free, long lasting HBeAg seroconversion, and also HBsAg negative status. Therefore, PEG-IFN monotherapy is recommended as the first choice treatment for CHB by not only the Japanese Society of Hepatology (JSH) but also the European Association for the Study of the Liver (EASL) [9], and the American Association for the Study of Liver Diseases (AASLD) [26]. In the present study, the triple response rate was 21 % (3/14) at 5 years after completion of PEG-IFN in HBeAg-positive patients. This rate is identical to that reported by our group in another study of HBeAg-positive patients [21 % (53/247)] at 5 years after the completion of treatment with conventional IFN [2]. Further studies of larger group of patients and longer posttreatment follow-up period are needed.

On the other hand, treatment with NA is effective in patients at high risk of progression to liver fibrosis and cirrhosis. We have determined the antiviral potency after 4 years of continuous entecavir treatment in patients with CHB infection, and reported that incremental increases were observed in the rates of undetectable HBV-DNA, HBeAg seroconversion, and ALT normalization, reaching 96 %, 38 %, and 93 %, respectively, by 4 years [7]. These rates are higher than those achieved after PEG-IFN treatment, but in general, the HBsAg negative rate is lower than that after PEG-IFN, only 0.2 %-5.1 % after 1–5 years of treatment [31, 32]. On the other hand, in our previous study, we reported that previous IFN therapy provided prior to NA therapy, was associated with HBsAg clearance in HBeAg-positive patients (HBsAg clearance rate ratio (95 % CI); 6.15 (1.69-22.4), *p* = 0.006 by multivariate analysis) [33]. In that study, HBsAg clearance was more likely to occur in patients with high baseline ALT levels. Considered together, the results of our previous and present studies suggest that introduction of PEG-IFN therapy before NA therapy could be effective.

Our study had one major limitation related to the small sample size. However, the study was unique in that all participating patients completed the 24 and 48 weeks PEG-IFN alfa-2a treatment without discontinuation. Another advantage of the study was it provided the first documentation of the long-term outcome after treatment with PEG-IFN alfa-2a in patients with CHB in Japan. Another study of larger patient sample is needed to validate our findings.

## Conclusion

In conclusion, we investigated the long-term effects of PEG-IFN alfa-2a therapy in Japanese patients. At 5 years after completion of such therapy, the triple response rate in HBeAg-positive patients and combined response rate in HBeAg-negative patients were 21 % (3/14) and 25 % (1/4), respectively. The triple response was noted in all three patients treated with PEG-IFN alfa-2a for 48 weeks. Further studies of larger sample size, longer duration of treatment (>1 year) and longer follow-up, are warranted in order to confirm the present findings and establish the true response to PEG-IFN alfa-2a therapy in Japan.

## Abbreviations

ALT: Alanine transferase
AST: Aspartate transaminase
CHB: Chronic hepatitis B
CLEIA: Chemiluminescent enzyme immunoassay
ELISA: Enzyme-linked immunosorbent assay
HBeAg: Hepatitis B e antigen
HBsAg: Hepatitis B surface antigen
HBsAb: Hepatitis B antibody
HBV: Hepatitis B virus
HCC: Hepatocellular carcinoma
IFN: Interferon
JSH: The Japanese Society of Hepatology
PCR: Polymerase chain reaction
PEG-IFN: Peginterferon
